# An Intersectional Perspective on Cyberbullying: Victimization Experiences Among Marginalized Youth

**DOI:** 10.1002/jad.12466

**Published:** 2025-01-18

**Authors:** Alberto Amadori, André Gonzales Real, Antonella Brighi, Stephen T. Russell

**Affiliations:** ^1^ Faculty of Education Free University of Bozen‐Bolzano Brixen‐Bressanone Italy; ^2^ Department of Human Development and Family Sciences The University of Texas at Austin Austin Texas USA

**Keywords:** adolescence, cyberbullying, gender modality, intersectionality, minorities, sexual orientation, victimization

## Abstract

**Introduction:**

The impact of cyberbullying victimization on youth development, encompassing mental health, academic performance, and socioemotional well‐being, has been widely documented. Research highlights the heightened vulnerability of sexual and gender minoritized youth, along with other youth from marginalized groups, to cybervictimization. However, there is a gap in understanding how intersecting marginalized social identities affect experiences of cyberbullying.

**Methods:**

This study employs an intersectionality framework to examine cybervictimization among youth. The sample consists of 444,224 students in grades 9–12 from the 2017–2019 California Healthy Kids Survey. Using exhaustive chi‐square automatic interaction detection (ECHAID), the analysis identifies the prevalence of cybervictimization across multiple intersecting social identities, including sex assigned at birth, gender modality (cisgender 97.6%), sexual orientation, race/ethnicity, grade level, and socioeconomic status.

**Results:**

Cybervictimization was reported by 22.7% of youth in the sample. Rates were two to three times higher among youth with multiple marginalized identities. Youth at the intersection of bisexual sexual orientation, transgender gender modality, and racial/ethnic minoritized identities faced a particularly high risk of cybervictimization.

**Conclusions:**

There is an urgent need for future research in cyberbullying and youth development. Such research should focus on identifying and understanding the intersectional nature of discrimination and victimization, both in‐person and online, to develop evidence‐based prevention programs that effectively address the complexities of minoritized identities and discrimination in the digital world.

Cybervictimization constitutes the experience of being targeted through digital channels for acts such as bullying, harassment, and humiliation, a phenomenon that encompasses socioeconomic, ethnic, and social stratifications (Ansary 2020), often culminating in emotional distress, harm, or other adverse outcomes. Addressing cybervictimization among youth is crucial due to its extensive impact on their well‐being and development, especially in today's digital age characterized by pervasive exposure. Recognizing the vulnerability of youth to emotional and psychological consequences (Chudal et al. 2022; Dorol‐Beauroy‐Eustache and Mishara 2021; Nixon 2014), as well as the potential long‐term negative effects on academic performance and social relationships (Gardella, Fisher, and Teurbe‐Tolon 2017; Kowalski et al. 2014; Wright 2015), it is important to understand and mitigate cybervictimization. Intersectionality theory is used to understand how various systems of oppression such as racism, sexism, and other forms of discrimination intersect and interact to exacerbate health disparities among youths (Bowleg 2012; Crenshaw 1991; Shields 2008). These systems of oppression are sociostructural and systemic, influencing the lived experiences of marginalized individuals. This is particularly relevant given the increasing diversity of youth populations (Van Hook et al. 2023). However, there is a gap in understanding how the intersection of multiple marginalized social identities is reflected in experiences of cybervictimization among youth. In this study, intersectionality serves as a critical analytic framework to examine how overlapping social identities and related systems of oppression shape the experiences of cybervictimization among youth.

Several studies have pointed to the heightened vulnerability of girls to cyberbullying victimization when compared to boys (Brighi et al. 2019; Smith et al. 2019; Wachs, Junger, and Sittichai 2015), particularly in the context of sexual victimization and body shaming (Aboujaoude et al. 2015). Other research, however, suggests that boys might have a higher risk of becoming cybervictims (Huang and Chou 2010), especially when they express traits typically associated with femininity (Wright and Wachs 2020). While it is hard to define a trend of cybervictimization between boys and girls, research indicates that transgender and gender diverse (TGD) youth emerge as a group uniquely vulnerable to cybervictimization (Eisenberg et al. 2017; Lian et al. 2022; Ojeda, Elipe, and Del Rey 2023). Studies demonstrated that TGD youth reported 2.9 times more instances of cybervictimization than their cisgender peers (Meyer et al. 2017), even surpassing the vulnerability levels observed among genderqueer youth (Garthe et al. 2021).

Further, sexually minoritized people tend to face greater vulnerability to online victimization (Elipe, de la Oliva Muñoz, and Del Rey 2018; Gámez‐Guadix and Incera 2021; Ojeda, Elipe, and Del Rey 2023; Wright and Wachs 2021). They frequently encounter individual stigma and a lack of safety at school, which contribute to a greater risk of cybervictimization (Strohmeier, Gradinger, and Yanagida 2022). Research shows that individual stigma and lack of safety at school are common experiences among sexually minoritized youth, and those factors also predict cybervictimization. Findings consistently show that gay/lesbian, bisexual, and youth questioning their sexual orientation are disproportionately subjected to cybervictimization in comparison to their heterosexual peers (Abreu and Kenny 2018; Angoff and Barnhart 2021; Bishop, Ioverno, and Russell 2023; DeSmet et al. 2018; Elipe, de la Oliva Muñoz, and Del Rey 2018; Meyer et al. 2017).

Existing research suggests that there are complex patterns in cybervictimization at the intersection of marginalized identities. For instance, sexual minority youth experience higher rates of cybervictimization when compared to heterosexuals, yet this disparity is concentrated among those assigned females at birth (Bishop, Ioverno, and Russell 2023). Additionally, research has shown that bisexual women within sexual minority groups experience more severe instances of adult sexual victimization and face higher rates of revictimization compared to others (Hequembourg and Dearing 2013; Salim, McConnell, and Messman 2023). The connection between sexual victimization and cybervictimization is notable, as the online environment can amplify the impact of sexual victimization, leading to increased harassment and cyberbullying. Bisexual women experience heightened susceptibility to cybervictimization, as their marginalized sexual identity makes them frequent targets of intersecting forms of sexual harassment and victimization (Flanders et al. 2017).

Existing studies have emphasized the disproportionate rates of cyberbullying victimization experienced by racial/ethnic minorities (Goebert et al. 2011; Sung Hong et al. 2016; Low and Espelage 2013; Stoll and Block 2015; Schneider et al. 2012). In a population study conducted more than a decade ago, Black students reported the highest rates of cyberbullying at 10.9%, followed by Latina/x/o students at 9.6%, individuals from other race/ethnic backgrounds at 7.3%, and White students at 6.7% (Wang, Iannotti, and Nansel 2009). A more recent review conducted by Hamm et al. (2015) suggested a discrepancy in the outcomes related to ethnic groups, with mixed or inconsistent outcomes between studies. Moreover, a recent longitudinal study examined rates of cyberbullying over time among youth and found that White participants reported more cyberbullying victimization over time compared to non‐White (Barlett and Wright 2018), suggesting that there may be variations in how victimization is perceived across different cultural contexts. Finally, North American studies on cyberbullying have not explored the experiences of minoritized groups such as American Indian/Native American or Multiracial individuals. Given mixed findings within race/ethnicity and cyberbullying research (Espinoza and Ismail 2021; Espinoza and Wright 2018), an intersectionality lens that incorporates race/ethnicity in combination with sex and sexual and gender modalities will contribute to an understanding of cyberbullying. In addition to gender, sexual orientation, and race/ethnicity, socioeconomic status (SES) is a significant factor influencing youth vulnerability to cyberbullying. Youth from economically disadvantaged backgrounds, may experience increased cyberbullying due to associated stigmatization, social exclusion, and limited access to supportive resources (Lee et al. 2023; Nagata et al. 2022; Tippett and Wolke 2014). Economic inequities can exacerbate feelings of marginalization, making these youth more susceptible to online victimization and compounding the adverse effects on their mental health and academic performance (Ronis and Slaunwhite 2019). Including SES as a dimension in this study allows for a more comprehensive understanding of how economic instability interacts with other marginalized identities in shaping cyberbullying experiences.

## 1 Intersectionality and Cyberbullying

Drawing on feminist theory (Bowleg 2012; Carbado et al. 2013; Crenshaw 1991), an intersectional analytical framework involves recognizing the complex interplay between individual experiences and social identities. This web of influences collectively molds an individual's lived reality in both online and offline spheres. Pioneering intersectional exploration by Stoll and Block (2015) demonstrated that power dynamics and social hierarchies play a crucial role in cyberbullying involvement. They found that students who belong to both racial/ethnic minority groups and nondominant gender modalities face unique challenges because they are subject to multiple layers of discrimination and prejudice.

## 2 The Present Study

As highlighted above, existing research has started to document that youth from marginalized social identities experience disproportionate rates of cybervictimization. However, these studies typically employed interaction effects (Stoll and Block 2015), multivariate logistic regressions (Angoff and Barnhart 2021), or multilevel analyses (Jensen et al. 2022), which are criticized for not capturing the complex interplay of social identities (Bauer 2014; del Río‐González, Holt, and Bowleg 2021). By utilizing a statistical approach recommended for quantitative research focusing on intersectionality (Bauer et al. 2021), we aim to gain a deeper understanding of how rates of cybervictimization vary across multiple intersecting identities that represent forms of oppression that interact to shape the experience of cybervictimization (Crooks 2016). In this study, we investigate the intersections of sex assigned at birth, gender modality, sexual orientation, race/ethnicity, and SES in association with the prevalence and patterns of cyberbullying victimization among adolescents.

## 3 Methods

### 3.1 Data and Sample

Data come from the 2017–2019 California Healthy Kids Survey (CHKS), an anonymous online or paper‐delivered survey administered to students in classrooms for grades 7, 9, and 11 from California public schools every other year in both English and Spanish (a small number of students in grades 10 and 12 attending those classes were included as participants). Participation rates varied between 56% and 77% depending on grade level. Approximately 2% of surveys were excluded because participants had highly unlikely responses or declared untruthful responses, and 7% of the surveys were excluded because some schools omitted questions on the social identities of interest. The present analysis includes 444,224 CHKS students from 9th to 12th grades, who were asked about experiences of cybervictimization and multiple social identities. More information on the CHKS can be found in other sources (WestEd 2019). The Office of Research Support and Compliance from the University of Texas at Austin deemed the study exempt from review as it did not constitute human subjects research, given that the analysis used existing anonymized data (FWA #00002030).

### 3.2 Survey Measures

#### 3.2.1 Social Identity

Five social identities were examined: sex assigned at birth, race/ethnicity, receipt of free lunch (used as a proxy for SES), gender modality, and sexual orientation. Gender modality captures the relationship between a participant's gender identity and their gender assigned at birth (e.g., cisgender, transgender, questioning), while gender identity reflects how participants self‐identify (e.g., boy, girl, nonbinary). To better understand participants' social positioning, our analysis categorized them based on their reported gender modality. Students provided information about their sex assigned at birth (male; female). Given developmental differences in cyberbullying (Evangelio et al. 2022), we include school grades as an additional social identity.

Race/ethnicity was assessed with two separate questions. Participants were asked: “Are you of Hispanic or Latino origin?” (Yes; No); those who answered “Yes” were coded as Latina/x/o. A separate question asked: “What is your race?” (American Indian or Alaska Native; Asian; Black or African American; Native Hawaiian or Pacific Islander; White; Multiracial). A combined measure of mutually exclusive categories was created. First, participants were coded as Latina/x/o regardless of their race. Next, participants' race responses were used to code non‐Latina/x/o (NL) racial groups (e.g., NL Black or African American; NL API—Asian or Pacific Islander).

As a measure of SES, participants reported whether they received free or reduced‐price lunch at school. When participants were not sure or did not know (11.34%), they were coded as missing (but retained in analyses as described below).

Gender modality was assessed with the question: “Some people describe themselves as transgender when their sex at birth does not match the way they think or feel about their gender. Are you transgender?” Responses were coded as transgender (Yes), cisgender (No), questioning (Not sure), or missing (Declined to respond). This approach categorizes participants based on their gender modality to capture experiences related to their relationship with the gender assigned at birth (Ashley 2022).

To describe their sexual orientation participants were asked: “Which of the following best describes you?” (Straight; Gay or lesbian; Bisexual; Uncertain; Other; Decline to respond). Those who declined to respond were coded as missing, while those uncertain about their sexual orientation were coded as “Questioning.”

#### 3.2.2 Cybervictimization Experience

To evaluate participants' encounters with cybervictimization, the survey included the following question: “During the past 12 months, how many times did other students spread mean rumors or lies, or hurtful pictures, about you online, on social media, or on a cell phone?” (Never; One time; two to three times; four or more times). Responses were dichotomized as categorical variables with values of 1 indicating any occurrences of cyberbullying victimization experiences in the past 12 months. Dichotomizing strengthens statistical power by addressing limited variance across frequency categories, aligning with research emphasizing any exposure's importance over frequency in assessing cybervictimization's impact on adolescent health outcomes (Gámez‐Guadix and Incera 2021).

### 3.3 Data Analysis

Data were managed using Stata 18.0 (StataCorp 2023) and analyzed using SPSS Version 27. An exhaustive *χ* ^2^ automatic interaction detection (ECHAID) with Bonferroni corrections was conducted (Kass 1980). Prior work examining intersectionality in large samples of youth has adopted this approach (Eisenberg et al. 2024; Gower et al. 2023). The ECHAID works as a decision‐tree approach allowing the examination of how cybervictimization experiences may vary among distinct intersections of social identities. This is accomplished by conducting multiple stepwise *χ* ^2^s tests (using a Bonferroni‐adjusted *p* value) to find which of the independent variables (i.e., social identities) exhibit the largest differentiation in relation to the dependent variable (i.e., prevalence of cybervictimization). Additionally, splits between categories that present significantly different prevalence on the dependent variable are generated. Categories further split where there are significant statistical differences between categories (*p* < 0.05) and terminate when no further splits are significant, the minimum group size is not met (> 100 participants to prevent overfitting), or the splitting process reaches the maximum depth of five branches. This process enables the identification of differences in the prevalence of cybervictimization among unique combinations of social identities. Importantly, the ECHAID analysis allows the incorporation of missing responses as a distinct category of the independent variables. We present 10 intersectional groups with the highest prevalence of cybervictimization.

## 4 Results

Approximately equal proportions of participants were assigned male at birth and assigned female at birth (Table 1). Most participants identified as Latina/x/o (51.5%). Most participants reported a cisgender gender modality and identified as heterosexual, while 12.8% of participants belonged to sexual and/or gender minoritized groups, including those with a transgender or questioning gender modality. Moreover, ~1 in every 5 participants (22.7%) reported cyberbullying victimization in the past 12 months.

**Table 1:** Sample descriptive statistics.

	*N* = 444,224	(%)
Grade		
9th	208,458	(46.9)
10th	26,721	(6.0)
11th	182,443	(41.1)
12th	26,602	(6.0)
Sex assigned at birth		
Male	217,243	(49.9)
Female	218,411	(50.1)
Missing	8570	(1.9)
Race and ethnicity		
NL Native American	3314	(0.8)
NL Asian/Pacific Islander	56,304	(12.8)
NL Black	15,227	(3.5)
Latina/x/o	224,357	(51.0)
NL White	102,386	(23.3)
NL Multiracial	38,519	(8.8)
Missing	4117	(0.9)
Sexual orientation		
Straight	364,416	(85.7)
Gay or lesbian	8457	(2.0)
Bisexual	28,305	(6.7)
Questioning	17,201	(4.0)
Something else^a^	7021	(1.7)
Missing	18,824	(4.2)
Gender modality		
Cisgender	416,806	(97.6)
Transgender	4269	(1.0)
Questioning	5840	(1.4)
Missing	17,309	(3.9)
Receipt of reduced‐priced/free lunch		
No	188,777	(48.1)
Yes	203,659	(51.9)
Missing	51,788	(11.7)
Cyberbullying		
No	343,535	(77.3)
Yes	100,689	(22.7)

*Note:* Percent of missing calculated based on full *N*. All valid response options are shown as the valid percent (excludes missing values).

Abbreviation: NL, non‐Latino.

^a^ Something else = includes participants who reported other sexual orientations, including queer or pansexual.

Table 2 illustrates the 10 intersectional groups with the highest prevalence of cyberbullying victimization in the past 12 months. For example, youth assigned male at birth, who reported being NL Asian or Pacific Islander, NL Black, or did not report race/ethnicity, and had a transgender or questioning gender modality, or did not disclose their gender modality, as well as gay or lesbian, and those who reported receipt of free lunch was the group with the highest prevalence of cybervictimization (63.0%).

**Table 2:** Results from top 10 highest prevalence nodes from exhaustive ECHAID for cyberbullying (22.7% overall).

Prevalence (%)	Sex assigned at birth	Race or ethnicity	Gender modality	Sexual orientation	Receipt of free lunch	Grade
63.0	Male	NL API/NL Black/missing	Questioning/TGD/missing	Lesbian/gay	Yes	—
52.0	Female	NL multiracial/NL AIAN	Questioning/missing	Bisexual	—	—
51.0	Missing	—	TGD	Lesbian/gay/bisexual/something else/questioning	—	—
45.1	Male	—	TGD	Something else	—	—
43.2	Male	NL Multiracial/Latina/x/o/non‐Latino AIAN	TGD/questioning/missing	Lesbian/gay	Yes	—
43.0	Male	—	TGD/questioning	Missing	—	9th or 10th
42.7	Male	—	TGD/questioning	Bisexual	—	9th or 10th
42.3	Female	NL White/Latina/x/o/missing	Questioning/missing	Bisexual	—	11th or 12th
42.2	Female	NL multiracial/NL AIAN	Cisgender/TGD	Bisexual	—	9th or 10th
41.3	Female	NL White/Latina/x/o/missing	—	Bisexual	—	9th or 10th

*Note:* Index: The prevalence of the node relative to the prevalence of the full sample. For cells that display “—,” nodes did not divide based on a specific social identity (e.g., Receipt of free lunch). These nodes include participants from all categories within that social identity (e.g., Yes, No, Missing).

Abbreviations: AIAN, American Indian or Alaskan Native; API, Asian or Pacific Islander; LG, lesbian or gay; NL, Non‐Latina/x/o; TGD, transgender and gender diverse.

Cybervictimization reports were most prevalent among youth who occupied multiple marginalized social positions, for whom it was two to three times more prevalent than the overall sample. Among the highest prevalence intersectional groups, youth with a transgender or questioning gender modality, those from racial/ethnic minoritized groups, in 9th or 10th grades, and those identifying as sexual minorities (particularly bisexuals), disproportionately reported cyberbullying victimization. The highest prevalence of cybervictimization was observed among youths who reported a combination of these social positions. More specifically, a pattern revealed that bisexual individuals assigned female at birth, belonging to racial/ethnic minority groups, in 9th or 10th grades, and having a transgender or questioning gender modality consistently characterized the groups that reported among the 10 highest rates of cybervictimization. For this analysis, we focused on presenting only the highest terminal nodes resulting from the ECHAID analysis. However, it's important to note that the lowest terminal nodes also showed a prevalence of cyberbullying, ranging from 13.5% to 18.1%. This indicates that cyberbullying is a widespread phenomenon among youth, regardless of specific social identities. An extended table, including both the highest and lowest terminal nodes, can be found in the Supporting Information.

## 5 Discussion

Literature on youth development underscores the significant impact of cyberbullying on adolescents' developmental trajectories and mental well‐being (Hu et al. 2021; Kowalski et al. 2014). This is especially relevant for marginalized groups, who face ongoing discrimination in digital spaces, a challenge that becomes particularly pronounced during adolescence. In this study, we examined how different social positions and multiple dimensions of various social identities shape intersectional experiences of cyberviolence among the youth. Three key themes emerged from our results that contribute to understanding cyberbullying through the lens of intersectionality: (1) the heightened vulnerability of youth occupying multiple marginalized identities to cyberbullying, (2) the role of digital platforms in both providing support and exposing youth to risk, and (3) the compounded impact of intersectional discrimination on mental health and well‐being.

The present study highlights that youth with non‐cisgender gender modalities, particularly those at the intersection of racial/ethnic minority status, sexual minority status, and lower socioeconomic backgrounds, are disproportionately impacted by cybervictimization. The concept of gender modality enriches this intersectional analysis by providing a framework to examine how relationships between assigned gender and gender identity shape experiences of online marginalization. Our use of gender modality rather than solely gender identity reflects the importance of capturing the broader relational dynamics of gender identity and assigned gender. Enhanced internet engagement among minoritized youth primarily reflects broader societal dynamics and discrimination experienced offline (Devito, Walker, Birnholtz 2018; Robinson et al. 2020; Kilvington 2021). Sexual and gender minoritized youth often use social media platforms as a source of information, identity exploration, and social support (Hiebert and Kortes‐Miller 2023; Saha, Chandrasekharan, and De Choudhury 2019). However, the increased online presence for these minoritized groups also might heighten their exposure to cyberbullying (Hillier, Mitchell, and Ybarra 2012; Ybarra and Mitchell 2016).

Sexual and gender minoritized youth, especially those from racial or ethnic minoritized backgrounds, are disproportionately affected by cybervictimization. This trend is most pronounced among youth with a transgender or questioning gender modality, with these groups experiencing notably higher rates of cyberbullying compared to their peers These vulnerabilities may not simply be the result of individual prejudice but are deeply rooted in a matrix of sociocultural norms that marginalize certain identities more than others. For example, sexually minoritized youths who are also part of racial or ethnic minoritized groups face a compounded form of discrimination known as intersectionality, where the effects of racism and homophobia (or transphobia) are not merely additive but multiplicative, creating a significantly heightened risk of cybervictimization. Schools and social platforms may lack the training or policies needed to address the unique vulnerabilities of youth with transgender or questioning gender modalities, particularly when intersecting with racial/ethnic minority status. Incorporating the concept of gender modality into training programs and policies can help create safer and more inclusive environments for these youth (Ashley, Brightly‐Brown, and Rider 2024). Additionally, structural inequities, such as socioeconomic disparities and limited access to mental health resources, hinder the ability of these youth to recover from or combat the effects of cyberbullying.

The lived experiences of cyberbullying among these youths vary widely. Youth with transgender or questioning gender modalities frequently encounter misgendering and targeted harassment, which is often rooted in societal discomfort with non‐cisgender modalities (Fosch‐Villaronga et al. 2021; Ingram 2019). Such experiences can result in profound psychological distress and withdrawal from online spaces, which are otherwise vital sources of support for marginalized groups. Racial/ethnic minority youths report experiencing racist slurs and exclusionary tactics that reinforce their social isolation (Tynes et al. 2019; Patchin and Hinduja 2020). Sexual minority youths, especially those who identify as bisexual, frequently encounter bi‐erasure and invalidation of their sexual identity from both heterosexual and other sexual minority peers (Feinstein et al. 2019; Serpe et al. 2020; Velasco, Miranda‐Tena, and Sanmartín 2024). These experiences are not only frequent but also intensely personal, often leading to severe emotional and mental health consequences. Youth who occupy multiple intersecting marginalized identities—such as bisexual individuals with transgender or questioning gender modalities from racial/ethnic minority backgrounds—may face an intensified form of cyberbullying, where the combined effects of racism, homophobia, and gender‐based discrimination create unique and compounded challenges (Bowleg 2012; Crenshaw 1991). These overlapping forms of discrimination are not simply additive but interact in ways that amplify the risk and impact of cybervictimization, leading to severe emotional and mental health consequences. Research highlights that youth with intersecting identities experience more profound social isolation and mental health struggles as a result of cyberbullying, which can be challenging to address within current school and platform policies (Robinson et al. 2020; Sterzing et al. 2017).

The combination of identifying as bisexual, being assigned female at birth, and having a transgender or questioning gender modality was prominent in 4 out of the 10 highest prevalence groups. These findings align with previous studies that show bisexual youth as having the highest odds of experiencing cybervictimization (Angoff and Barnhart 2021; Ojeda, Elipe, and Del Rey 2023). Bisexual youth encounter stigma because they challenge both heterosexual and monosexual norms (Feinstein et al. 2019), which may contribute to a dual stigma and heightened susceptibility to cyberbullying experiences (Dodge 2021; Flanders et al. 2017; Molina et al. 2015; Taylor et al. 2019). These results may help explain, for example, evidence that bisexual youth are at a greater risk of mental health challenges compared to other sexual minoritized groups (Friedman et al. 2011; Marshal et al. 2011; Saewyc et al. 2006). Importantly, our results extend the growing understanding of bisexual youth well‐being by identifying that the greatest burden of cyberbullying is specifically among bisexual assigned female at birth who also identify as gender and/or racial/ethnic minorities.

Consistent with previous research (Kosciw et al. 2020; Ojeda, Elipe, and Del Rey 2023), our analysis yielded elevated rates of cybervictimization among TGD youths. Research consistently shows that TGD youths, for whom behavior and expression are often inconsistent with traditional gender norms, face greater cybervictimization due to societal discrimination exacerbated by online anonymity (Johns et al. 2019; Norris and Orchowski 2019; Sterzing et al. 2017). These intersectional pressures create high‐risk environments for these adolescents in digital spaces, leading to experiences of sustained harassment, threats of violence, and derogatory comments that can severely impact their mental health and academic performance.

Aligning with previous findings, our results indicate that SES significantly influences youth vulnerability to cyberbullying (Lee et al. 2023; Tippett and Wolke 2014). Youth from economically disadvantaged backgrounds experience heightened cyberbullying due to factors such as stigmatization, social exclusion, and limited access to supportive resources (Nagata et al. 2022). This suggests that economic inequities intensify feelings of marginalization, making these youth more susceptible to online victimization and amplifying the adverse effects on mental health and academic performance (Ronis and Slaunwhite 2019). Future research should examine the specific intersectional pathways through which economic instability exacerbates cyberbullying risks, including the potential moderating effects of social support networks and digital literacy interventions in reducing these adverse outcomes.

### 5.1 Strengths and Limitations

This study has several notable strengths and limitations. We use an extensive and diverse sample, allowing analyses that illuminate complex intersections for small prevalence groups. This study's strength lies in its employment of intersectional analysis, which accounts for the combination of social identities and their potential influence on vulnerability and impact. However, there are several limitations. First, sole reliance on a single‐item measure for cybervictimization risks compromising psychometric accuracy (Diamantopoulos et al. 2012), and overlooking the multifaceted nature of online bullying, which often encompasses acts like doxing and identity theft. Second, using the receipt of free lunch as the sole indicator of SES may oversimplify its multidimensional nature. Third, the use of ECHAID can result in groupings or artificial splits that may not generalize beyond the sample (Bauer et al. 2021; Gower et al. 2023). Additionally, our choice to treat Latino/a/x as mutually exclusive of other racial identities limits the ability to understand the experiences of Black vs. White Latine youth. This choice restricts insight into how intersecting racial and ethnic identities uniquely shape cybervictimization experiences, which could be particularly relevant for understanding how compounded discrimination manifests online. Moreover, while our use of gender modality provides valuable insights, it may oversimplify individual identities by categorizing participants into broad modalities. Future research should integrate both gender identity and modality to better reflect participants’ lived experiences. Lastly, we were limited to using grade level as a proxy for age, which may not accurately reflect developmental differences among students of the same grade but varying ages. This approach could obscure subtle age‐related variations in cyberbullying experiences, as age rather than grade may better represent maturity and developmental stage, impacting vulnerability and coping mechanisms for cybervictimization.

### 5.2 Implications and Conclusions

Our findings underscore a critical nexus between cyberbullying and intersectional identities, highlighting the disproportionate impact on minoritized youth. This emphasizes the need for tailored online safety measures and educational strategies that address the specific vulnerabilities of sexual and gender minorities, especially within racial and ethnic groups (Amadori et al. 2023; Eisenberg et al. 2024). Schools and online platforms must adopt targeted interventions that are informed by intersectional insights to effectively combat cyberbullying. Implementing inclusive policies and diversity training can significantly mitigate bias‐based bullying, promoting safer and more supportive environments for all youth.

## Ethics Statement

This study has been determined by the Office of Research Support (ORS) to not involve human subjects research, as per FWA #00002030. The activities conducted in this research do not meet the criteria for human subjects research as defined in the Common Rule (45 CFR 46) or FDA Regulations (21 CFR 56). IRB review and oversight are not required as the study does not involve human interactions, but rather falls under categories such as classroom activities, program evaluation, secondary use of de‐identified data, obtaining publicly available information, biographical research not generalizable beyond the individual, archival research, or other activities specified in the determination letter dated.

## Conflicts of Interest

The authors declare no conflicts of interest.

## Supporting information

Supporting information.

## Data Availability

The authors do not have permission to share data.
