# Supplementary material for: An Intersectional Perspective on Cyberbullying: Victimization Experiences Among Marginalized Youth
Source: J Adolesc. 2025 Jan 18;97(4):931–40. doi: 10.1002/jad.12466 (PMC12128902; doi:10.1002/jad.12466)
Supplement: Supplementary file 1 — Supporting information. [file JAD-97-931-s001.docx]

**Table**

*Results From Lowest 10 Bottom and Top 10 Highest Prevalence Nodes From Exhaustive ECHAID for Cyberbullying (22.7% Overall).*

| Prevalence (%) | Sex Assigned at Birth | Race or Ethnicity | Gender Modality | Sexual Orientation | Receipt of Free Lunch | Grade |
| --- | --- | --- | --- | --- | --- | --- |
| *Lowest ten* | |  |  |  |  |  |
| 13.5% | Male | NL API | Cisgender | Straight | Yes/ Missing | -- |
| 14.2% | Male | NL API/Missing | Cisgender/Missing | Missing | Yes/Missing | -- |
| 15.3% | Male | NL API | Cisgender/Missing | Something else | -- | -- |
| 15.6% | Male | NL API | Cisgender | Straight | No | -- |
| 16.2% | Male | NL API/Latina/x/o/Missing | Missing | Straight | -- | -- |
| 17.0% | Male | NL Multiracial/ NL White/ NL Black/ NL AIAN | Cisgender | Straight | -- | 11^th^ or 12^th^ |
| 17.0% | Male | -- | Cisgender | Questioning | Missing | -- |
| 17.4% | Female | NL API/Missing | -- | Missing | -- | -- |
| 17.4% | Missing | -- | -- | Straight/Missing | Yes/Missing | 11^th^ or 12^th^ |
| 18.1% | Male | NL Multiracial/Non-Latino White/Non-Latino Black/Non-Latino AIAN | Cisgender/Missing | Missing | Yes/Missing | -- |
| *Highest ten* | |  |  |  |  |  |
| 63.0% | Male | NL API/Non-Latino Black/Missing | Questioning/TGD/Missing | Lesbian/Gay | Yes | -- |
| 52.0% | Female | NL Multiracial/Non-Latino AIAN | Questioning/Missing | Bisexual | -- | -- |
| 51.0% | Missing | -- | TGD | Lesbian/Gay/Bisexual/Something else/Questioning | -- | -- |
| 45.1% | Male | -- | TGD | Something else | -- | -- |
| 43.2% | Male | NL Multiracial/Latina/x/o/ NL AIAN | TGD/Questioning/Missing | Lesbian/Gay | Yes | -- |
| 43.0% | Male | -- | TGD/Questioning | Missing | -- | 9th or 10th |
| 42.7% | Male | -- | TGD/Questioning | Bisexual | -- | 9th or 10th |
| 42.3% | Female | NL White/Latina/x/o/Missing | Questioning/Missing | Bisexual | -- | 11th or 12th |
| 42.2% | Female | NL Multiracial/ NL AIAN | Cisgender/TGD | Bisexual | -- | 9th or 10th |
| 41.3% | Female | NL White/Latina/x/o/Missing | -- | Bisexual | -- | 9th or 10th |

*Note:* Index: the prevalence of the node relative to the prevalence of the full sample. For cells that display "--", nodes did not divide based on a specific social position (e.g., Receipt of free lunch). These nodes include participants from all categories within that social position (e.g., Yes, No, Missing). NL: Non-Latina/x/o. TGD: Transgender and gender diverse. API: Asian or Pacific Islander. AIAN: American Indian or Alaskan Native. LG: Lesbian or Gay.
